# Prevalence of Emotional Factors and Pain in Temporomandibular Disorder and Correlation With Different Diagnoses: A Cross‐Sectional Study

**DOI:** 10.1111/joor.70084

**Published:** 2025-10-15

**Authors:** Gabriela Caovilla Felin, Cassiano Mateus Forcelini, Alvaro Della Bona

**Affiliations:** ^1^ Post‐Graduate Program in Dentistry Universidade de Passo Fundo/RS Passo Fundo RS Brazil

**Keywords:** anxiety, depression, observational study, pain, temporomandibular joint disorders

## Abstract

**Background:**

Temporomandibular disorder (TMD) is a leading cause of orofacial pain, often associated with psychosocial factors that affect quality of life. Although these factors are well documented, the association with pain intensity and jaw function remains understudied.

**Objective:**

This cross‐sectional observational study aimed to report the prevalence of emotional factors in patients with TMD and to correlate TMD with psychological factors, pain intensity and functional limitation of the jaw in a sample of the regional population.

**Methods:**

This study used data from a TMD outpatient clinic in South America. Assessments included the DC‐TMD, chronic pain scale, GAD‐7 (anxiety), PHQ‐9 (depression), PHQ‐15 (somatic symptoms) and JFLS‐8 (jaw function) instruments. Sociodemographic data and the diagnosis of TMD were also reported. Data were statistically analysed using Kolmogorov–Smirnov, two‐tailed chi‐square, Fisher's exact, Mann–Whitney *U*, Kruskal–Wallis and Spearman's correlation coefficient tests (*α* = 0.05).

**Results:**

From 190 patients (mean age 43.5), 78.4% were women, 31.5% had low education and 44.7% had a household income lower than US$ 500. The majority had TMD with pain and joint disorder. The overall chronic pain score was 63, indicating high pain intensity. Psychosocial scores and jaw limitations were mild. Women showed higher pain levels, more functional limitations, and elevated psychosocial scores.

**Conclusion:**

Psychological factors did not differ significantly across TMD subtypes but were highly prevalent, especially in women.

## Graphical abstract

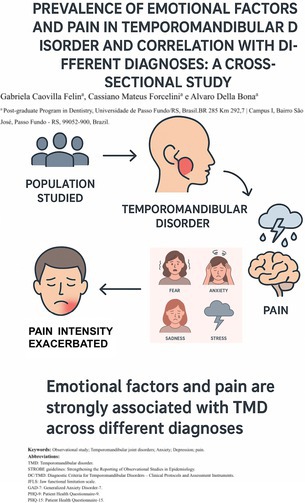

**Abbreviations**
DC/TMD: Diagnostic Criteria for Temporomandibular Disorders—Clinical Protocols and Assessment Instruments
GAD‐7: Generalised Anxiety Disorder‐7
JFLS: Jaw functional limitation scale
PHQ‐15: Patient Health Questionnaire‐15
PHQ‐9: Patient Health Questionnaire‐9
STROBE guidelines: Strengthening the Reporting of Observational Studies in Epidemiology
TMD: Temporomandibular disorder

## 1 Introduction

Temporomandibular disorder (TMD) is one of the primary causes of pain in the orofacial region, and its diagnosis has become increasingly frequent, mainly among individuals with psychosocial factors, adversely affecting their quality of life [1]. Given the numerous potential etiologies of pain, anatomical complexity and psychosocial impact, identifying and characterising the origin of pain has posed some challenges for healthcare workers [2].

Studies have demonstrated that psychological factors play a significant role in the duration and persistence of symptoms in cases of chronic TMD pain, and some of these factors have been identified as predictors for TMD [3]. The Diagnostic Criteria for Temporomandibular Disorders (DC/TMD) were designed to standardise clinical examination, diagnosis and classification of TMD subtypes, and they are widely used in clinical research. The DC/TMD are highly appreciated in clinical research because of their well‐documented and standardised specifications for conducting a systematic clinical examination of TMD and because of their dual‐axis approach, which records clinical, behavioural and psychosocial data [4, 5].

Systematic reviews provide the general prevalence of depression and anxiety in patients with TMD, but the studies included in such reviews differ widely among themselves and they also lack key data to establish any correlation [6, 7]. Therefore, further studies based on the DC/TMD recommendations are needed, considering that they better integrate both axes I and II from DC/TMD and further elucidate the aetiology of TMD [7]. Moreover, the prevalence of psychological factors such as anxiety and depression in patients with TMD is underreported in the South America population, and there is only a very short knowledge on what TMD subdiagnosis is most frequently associated with these factors.

There are recommendations for studies to correlate these factors and, therefore, achieve more effective and definitive treatments for TMDs [8, 9]. The aim of this study was to evaluate the prevalence of emotional factors and pain in patients with temporomandibular joint disorders (TMDs) and to correlate single and multiple TMD diagnoses with psychological factors, pain intensity and functional limitation of the jaw in a regional population sample, testing the hypothesis that there is a positive correlation between psychological factors and TMD diagnoses for individuals in the evaluated population sample.

## 2 Methods

### 2.1 Study Overview

The study followed some of the Strengthening the Reporting of Observational Studies in Epidemiology (STROBE) guidelines [10]. The study protocol was approved by the Research Ethics Committee from the local University (process no. 3.855.911), and all participants signed an informed consent form prior to their participation in the study.

### 2.2 Participants

Considering that approximately 50% of the population presents some sign or symptom of TMD [11], the following parameters were considered for sample calculation: a significance level of 5% and a power of 95%, resulting in at least 130 patients evaluated by the DC/TMD instrument.

This cross‐sectional study included 195 patients with TMD recruited between September 2019 and March 2023. The study used a prospective database from a TMD outpatient clinic affiliated with the Oral Maxillofacial Surgery and Traumatology Residency Program at the Dental School of a University in southern Brazil.

### 2.3 Data Collection

This study included patients over 18 years of age who were treated between September 2019 and March 2023 at a TMD clinic. Patients who did not authorise the use of their data, had incomplete data in their medical records, or had been diagnosed with bruxism were excluded from the study.

Data were collected from questionnaires (chronic pain scale, GAD‐7, PHQ‐9, PHQ‐15 and JFLS‐8) applied to the participants and from their records in compliance with the DC/TMD [12]. The diagnosis of TMD was based on DC/TMD axes I and II, according to which patients are classified as having disorders associated with pain, intra‐articular disorders or degenerative joint disorders. The diagnosis was confirmed using imaging exams (panoramic radiographs, magnetic resonance imaging or computed tomography). For better correlation across the different types of TMD, disorders were categorised into three groups: (1) disorder associated with pain, (2) intra‐articular disorders and (3) disorder associated with pain and intra‐articular disorders. The following sociodemographic variables were obtained from patient records: sex (male or female), self‐reported skin colour (white, black, brown, Asian or indigenous), age (completed age), education (years of schooling) and household income (in Brazilian currency).

### 2.4 Assessment of Pain Intensity

Characteristic pain intensity (CPI) was measured on a chronic pain scale consisting of eight items. Only the first question and the items 2–4 were used, considering the fact that the objective of the study was to evaluate pain intensity. The questions were: ‘How do you rate your facial pain at the moment?; What is the intensity of your worst pain in the last 30 days; and in the last 30 days, on average, what was your worst pain?’. The scale values were from 0 to 10, where 0 meant ‘no pain’ and 10 indicated ‘the worst possible pain’. The first question on the Graded Chronic Pain Scale was used to determine how many days in the last 6 months the individual had experienced orofacial pain. Items 2 to 4 were averaged and then multiplied by 10 to check whether pain intensity was low intensity pain, without disability (CPI < 50) or high intensity pain, without disability (CPI ≥ 50) [13].

### 2.5 Psychological Instruments and Jaw Functional Limitation Scale

Data related to psychological factors were collected using the Generalised Anxiety Disorder‐7 questionnaire (GAD‐7), with seven questions about the frequency at which the individual was bothered by problems such as nervousness, anxiety, irritability, among others. The level of anxiety was estimated using scores 0, 1, 2 and 3, which respectively denoted ‘never’, ‘many days’, ‘more than half the days’, and ‘almost every day’. The overall GAD‐7 score ranged from 0 to 21. Scores 5, 10 and 15 were regarded as cutoff points for mild, moderate and severe anxiety, respectively [14]. The nine‐item Patient Health Questionnaire (PHQ‐9) was used to assess depression, and the answers were classified into ‘never’, ‘many days’, ‘more than half the days’ and ‘almost every day’ (scores 0, 1, 2 and 3, respectively). The final PHQ‐9 score ranged from 0 to 27, and mild, moderate, moderately severe and severe depression were scored as 5, 10, 15 and 20, respectively [15]. Somatic symptoms associated were assessed by the 15‐item Patient Health Questionnaire (PHQ‐15), in which scores 0, 1 and 2 indicated ‘not bothered at all’, ‘slightly bothered’ and ‘deeply bothered’, respectively. Final scores 5, 10 and 15 denote cutoff points for low, intermediate and high severity of somatic symptoms, respectively [16].

The 8‐item version of the Jaw Functional Limitation Scale (JFLS‐8) was used in this study and consists of a questionnaire that assesses limitation in mastication, vertical jaw mobility and verbal and emotional expression, in which respondents rate their answers on an 11‐point scale (0–10), where 0 indicates no limitation and 10 represents severe limitation [17, 18].

### 2.6 Statistical Analysis

Data were double entered and cross‐checked in a Microsoft Excel 2016 database. Categorical data were described as percentages. Continuous quantitative variables were subjected to the Kolmogorov–Smirnov test to assess data distribution. The variables were asymmetrically distributed; therefore, they were presented as medians and interquartile ranges. A significance level of 5% (*p* = 0.05) was used for the statistical tests. The two‐tailed chi‐square test or Fisher's exact test, whenever appropriate, was used to compare the proportion of categorical variables between groups. The Mann–Whitney *U* test was employed to compare the medians between men and women, whereas the Kruskal–Wallis test was used to compare the clinical groups of TMD. Spearman's correlation coefficient was used for the simple correlation between continuous variables and the pain analog scale and the parafunction scale. The statistical analysis was performed using IBM SPSS Statistics 24.

## 3 Results

A total of 190 patients participated in the study. The median age was 43.5 years old (31–58 years old) and 78.4% of the patients were female, 81.5% were white, 31.5% had a low level of education, and 44.7% earned less than U$ 500 dollars per month (Table 1). As for the number of days with orofacial pain in the past 6 months, 78.9% reported pain for more than 30 days.

**TABLE 1:** Demographic characteristics of the sample (*n* = 190). Categorical variables are presented as percentages. Continuous variables are presented as medians and interquartile ranges [IQR].

Characteristic	Result
Female	78.4% (*n* = 149)
White	81.5% (*n* = 155)
Age (in years)	43.5 [31–58]
Not living with a partner	58.9% (*n* = 112)
Low level of education^a^	31.5% (*n* = 60)
Monthly household income (U$)	500 [320–640]
Monthly household income less than U$ 500	44.7% (*n* = 85)
Persistence of pain for more than 30 days	78.9% (*n* = 150)
TMD P^b^	31.0% (*n* = 59)
TMD A^c^	10.5% (*n* = 20)
TMD *P* + A^d^	58.4% (*n* = 111)

^a^ Up to 8 years of schooling.

^b^ TMD P: Disorder associated with pain.

^c^ TMD A: Intra‐articular disorders.

^d^ TMD *P* + A: Disorder associated with pain and with intra‐articular disorder.

Most patients had more than one diagnosis of TMD: 70.5% had local myalgia; 74.7% complained of referred myofascial pain; 57.3% presented with arthralgia; 22.1% reported headache related to TMD; 66.8% presented disc displacement (56.8% with reduction and 10% without reduction) and 5.2% presented degenerative joint disorder (Table 2).

**TABLE 2:** Clinical characteristics of the sample (*n* = 190). Continuous variables presented as medians and interquartile ranges [IQR]. Categorical variables presented as percentages.

Characteristic	Result
Chronic pain scale	63 [47–80]
Jaw functional limitation scale	2.5 [1.3–4.8]
Anxiety score	9 [4–14]
Depression score	6 [3–12]
Somatic symptoms score	7.5 [4.7–11.2]
Local myalgia	70,5% (*n* = 134)
Referred myofascial pain	74,7% (*n* = 142)
Arthralgia	57.3% (*n* = 109)
Bilateral	26.8% (*n* = 51)
Unilateral	30.5% (*n* = 58)
Headache attributable to TMD	22.1% (*n* = 42)
Disc displacement	66.8% (*n* = 127)
Disc displacement with reduction	56.8% (*n* = 108)
Disc displacement without reduction	10.0% (*n* = 19)
Degenerative joint disorder	5.2% (*n* = 10)
TMD clinical groups	
Disorder associated with pain	31.0% (*n* = 59)
Intra‐articular disorder	10.5% (*n* = 20)
Disorder associated with pain and with intra‐articular disorder	58.4% (*n* = 111)

Abbreviation: TMD, temporomandibular disorder.

Considering the psychological factors in the entire sample, the anxiety and depression score was low, as well as the severity of somatic symptoms (Table 2). The overall chronic pain score was 63, indicating high pain intensity. The jaw functional limitation scale averaged 2.5, indicating low chewing and mobility limitations (Table 2).

Psychosocial factors yielded different results for men and women. Anxiety was more frequent among women (*p* < 0.001), with a mean score of 10 (moderate anxiety). Depression also affected more women than men (*p* < 0.001), similarly to that observed for the score of somatic symptoms (*p* < 0.001) (Table 3). In the overall sample, 70.5% presented some degree of anxiety, 62.1% had some degree of depression, and 72.1% had some degree of somatic symptoms.

**TABLE 3:** Comparison of clinical characteristics and diagnosis between men and women (*n* = 190). Categorical variables are presented as percentages. Continuous variables are presented as medians and interquartile ranges [IQR].

Characteristic	Female (*n* = 149)	Male (*n* = 41)	*p*
Age (in years)	46 [32.0–58.0]	42 [26.5–56.5]	0.450
Household income (U$)	440 [300–720]	600 [400–600]	0.471
Chronic pain scale	63 [53–80]	43 [25.0–63.0]	< 0.001*
Jaw functional limitation scale	2.8 [1.8–4.9]	0.8 [0.2–4.1]	0.002*
Anxiety score	10 [4.5–15]	5 [3–8.5]	< 0.001*
Depression score	7 [4–12]	3 [0–6]	< 0.001*
Somatic symptoms score	8 [5–12]	5 [3–8]	< 0.001*
Local myalgia	74.5%	56.1%	0.032*
Referred myofascial pain	79.2%	58.5%	0.014*
Arthralgia	61.7%	41.5%	0.032*
Headache attributable to TMD^a^	26.8%	4.9%	0.002*
Disc displacement	63.8%	78.0%	0.095
Disc displacement with reduction (*n* = 108)	82.1%	93.8%	0.154
Disc displacement without reduction (*n* = 19)	17.9%	6.3%	0.154
Degenerative joint disorder	5.4%	4.9%	1.000
TMD clinical groups			0.519
TMD P^b^	29.9%	39.5%	
TMD A^c^	10.9%	10.5%	
TMD *P* + A^d^	59.2%	50.0%	

*Note:* Medians and interquartile ranges compared by the Mann–Whitney *U* test. Percentages compared with chi‐square test or, whenever appropriate, with Fisher's exact test (two‐tailed).

^a^ Temporomandibular disorder.

^b^ TMD P: Disorder associated with pain.

^c^ TMD A: Intra‐articular disorders.

^d^ TMD *P* + A: Disorder associated with pain and with intra‐articular disorder.

* Significant difference.

With respect to the various diagnoses of TMD, a significant difference was observed between men and women in the diagnosis of disorders associated with pain, with a higher prevalence for women (Table 3).

When comparing the groups with different diagnoses of TMD and clinical characteristics, no significant difference was found between them. No significant correlations were identified comparing the correlation coefficient of the chronic pain scale and functional limitation with other variables. A higher median age (*p* = 0.044) was observed in patients diagnosed with joint disorder and disorder associated with pain (Table 4).

**TABLE 4:** Comparison of clinical characteristics among TMD clinical groups (*n* = 190). Categorical variables are presented as percentages. Continuous variables are presented as medians and interquartile ranges [IQR].

Characteristic	TMD P^a^ (*n* = 59)	TMD A^b^ (*n* = 20)	TMD *P* + A^c^ (*n* = 111)	*p*
Age (in years)	47 [34–56]	36 [23.2–39]	46 [31–60]	0.044*
Female	74.6%	80%	82%	0.522
White	81.4%	85%	84.7%	0.843
Low level of education^d^	28.8%	25%	36%	0.471
Household income (U$)	410 [300–600]	420 [380–670]	500 [322–600]	0.872
Chronic pain scale	63 [47–83]	61.5 [45.5–68.2]	63 [47–80]	0.530
Jaw functional limitation scale	2.8 [1.6–5.3]	2.9 [0.8–5.3]	2.5 [1.3–4.5]	0.683
Anxiety score	10 [4–14]	8 [4–14.2]	9 [4–14]	0.368
Depression score	5 [3–12]	6 [4–12.5]	6 [3–12]	0.993
Somatic symptoms score	9 [5–13]	6 [5–8.7]	8 [4–11]	0.838

*Note:* Medians and interquartile ranges compared with the Kruskal–Wallis test. Percentages compared with two‐tailed chi‐square test.

^a^ TMD P: Disorder associated with pain.

^b^ TMD A: Intra‐articular disorders.

^c^ TMD *P* + A: Disorder associated with pain and with intra‐articular disorder.

^d^ Up to 8 years of schooling.

* Significant difference.

A weak to moderate, but significant, correlation was found in comparing the correlation coefficient of the chronic pain scale and functional limitation with other variables (Table 5).

**TABLE 5:** Spearman's rank correlation coefficient of graded chronic pain scores and jaw functional limitation scale with other continuous variables (*n* = 190).

Correlation	*r*	*p*	Interpretation of correlation
Graded chronic pain			
Age	0.228	0.002*	Weak
Anxiety score	0.387	< 0.001*	Weak
Depression score	0.306	≤ 0.001*	Weak
Somatic symptoms score	0.353	≤ 0.001*	Weak
Jaw functional limitation scale	0.626	≤ 0.001*	Moderate
Jaw functional limitation scale			
Age	0.257	0.083	None
Anxiety score	0.328	≤ 0.001*	Weak
Depression score	0.223	0.002*	Weak
Somatic symptoms score	0.298	≤ 0.001*	Weak

* Significant correlation.

## 4 Discussion

This cross‐sectional observational study used known diagnostic criteria to provide detailed information on a regional population with TMD and its correlation with some related factors.

In this study, most patients were female, as observed in previous studies [1, 19, 20]. Biological (e.g., hormonal), behavioural, and/or social factors (e.g., psychological factors) may play an important role, representing twice the risk of developing TMD in women compared to men. Women also seek treatment more often, which contributes to the higher prevalence of TMD in the population [21].

The analysis of psychosocial factors revealed that most patients presented some level of anxiety, depression and/or somatic symptoms, partially confirming the hypothesis of our study. Nevertheless, the overall score for these factors was mild for anxiety and depression and low for somatic symptoms. Limited scientific evidence exists on TMD associated with somatisation and psychosocial factors in the population of South America [6, 7]. The findings of the present study are expected to enhance the understanding of these factors. Despite prospective and retrospective studies with limited information [22], the data herein suggest that psychological variables are potential etiologic risk factors for TMD.

The most prevalent diagnosis of TMD in the present study was a disorder associated with pain and concurrent with joint disorder, followed by a disorder associated with pain and joint disorder alone. These findings are in agreement with previous studies that suggested myalgia as a common diagnosis among TMDs associated with pain, which is usually followed by joint disorder [7, 23, 24]. It was reported that the higher rates of muscle disorders are due to protocol inconsistencies regarding clinical palpation and that the updated DC/TMD, used in the present study, offers a more robust diagnostic approach, reducing possible false‐positive results, such as fatigue or post‐exercise sensitivity [25]. According to Valesan et al., few studies show the prevalence of TMD using the DC/TMD and there is a large population variability across different locations and regions, which explains the discrepant findings and underscores the importance of depicting the actual prevalence in the general population [26].

When the TMD diagnostic groups were analysed separately (single diagnosis), no significant difference was found among them. When anxiety scores alone were assessed, moderate anxiety was more prevalent in the group of disorders associated with pain, but with no significant difference. This finding is not in line with some studies [27, 28, 29]. Yet, while some studies indicated significant psychological differences among patients with some type of disorder, other studies failed to observe such findings. This inconsistency may be attributed, to some extent, to the lack of standardised diagnostic criteria and to the selected approach to evaluate the psychological factors [30, 31]. In addition, Kmeid et al. discussed the association between these psychological factors and the increase in parafunctional habits, suggesting that such habits can elevate muscle sensitivity and pain perception, potentially aggravating awake or sleep bruxism. This highlights the need to distinguish between parafunctional habits and TMD, indicating that parafunctional habits may have a stronger correlation with these factors [23]. Souza et al. reported an increase in cortisol release in catastrophic patients, leading to an abnormal adrenocortical response to pain, potentially increasing their susceptibility to chronic pain and either sustaining or intensifying their pain experience [19].

The present study also showed a weak correlation between pain intensity and functional jaw limitation and psychological factors. However, significant changes in jaw function and pain intensity were observed, suggesting that pain intensity was exacerbated by psychological factors and that these factors also aggravated functional jaw limitation. This finding could be attributed to the fact that the patients assessed had mild to moderate TMD. It has been suggested that TMD can affect some aspects of social functioning, such as communication or verbal expression, but not activities related to chewing and jaw mobility [32]. This is an important topic for future research.

Psychological factors differed between men and women. Anxiety, depression and somatic symptoms were more frequently observed in women. As for the different diagnoses of TMD, a significant difference between men and women was observed in the diagnosis of disorders associated with pain, showing a higher prevalence among women [33]. A systematic review and meta‐analysis conducted in 2022 reported that depression and anxiety affect women more often than men [7]. It is important to consider that self‐reported general health status, general chronic pain disorders, study location, age, ethnicity and psychosocial and genetic factors can influence and trigger different signs and symptoms of TMD, which should always be assessed [34].

An additional factor concerning the higher diagnosis of disorders associated with pain is that women are more likely than men to transition from acute to chronic pain, and this is related to psychological characteristics that differ between men and women and to the biological effects of female reproductive hormones [35]. Fluctuating levels of female sex hormones during puberty, pregnancy and menopause are associated with both TMD pain and temporomandibular joint degeneration, with oestrogen, for instance, contributing to the degeneration of the condylar cartilage and having, at the same time, a protective role for the subchondral bone [36]. Yap et al. found that male patients with TMD are prone to pain‐related conditions, whereas female patients seem to be more susceptible to a combination of conditions associated with pain and intra‐articular disorders. Their finding underscores the importance of examining sex‐related generational diversities in TMD subtypes to guide care and future research on the topic [37].

## 5 Conclusion

Psychological factors do not seem to influence TMD subdiagnoses for the assessed individuals, and they do not appear to increase pain intensity and jaw functional limitation. Yet, the prevalence of these factors in the overall population sample was high, with most individuals exhibiting some degree of anxiety, depression and somatic symptoms. Female subjects showed greater pain intensity and jaw functional limitation, as well as higher scores for psychosocial factors than men.

## Author Contributions

The authors declare that all authors participated in the study. The final version of the work was read and approved by all authors. Gabriela Caovilla Felin: Substantially contributed to the conception, planning, execution, data collection, analysis or interpretation of the work. Participated in the writing or critical review of the text. Approved the final version to be submitted. Agrees to take public responsibility for the content. Cassiano Forcelini: Substantially contributed to the statistical analysis and interpretation of the work. Participated in the writing or critical review of the text. Approved the final version to be submitted. Agrees to take public responsibility for the content. Alvaro Della Bona: Substantially contributed to the conception, planning, execution, data collection, analysis or interpretation of the work. Work advisor. Participated in the writing or critical review of the text. Approved the final version to be submitted. Agrees to take public responsibility for the content.

## Ethics Statement

The study protocol was approved by the local research ethics committee (process no. 3.855.911—CEP UPF/Plataforma Brasil).

## Conflicts of Interest

The authors declare no conflicts of interest.

## Data Availability

The authors declare that the data will be made available by the authors upon request. The data collection link is as follows: https://docs.google.com/spreadsheets/d/1NO9MdaPu0u‐85rkyHN‐GGfMa9NMXcLlE/edit?usp=sharin&ouid=115665301193605804389&rtpof=true&sd=true.
